# Supplementary material for: Signature of spin-phonon coupling driven charge density wave in a kagome magnet
Source: Nat Commun. 2023 Oct 4;14:6183. doi: 10.1038/s41467-023-41957-5 (PMC10550957; doi:10.1038/s41467-023-41957-5)
Supplement: Supplementary file 1 — Supplementary Info [file 41467_2023_41957_MOESM1_ESM.pdf]

Supplementary Information for

Signature of Spin-Phonon Coupling Driven Charge Density Wave in a Kagome Magnet

H. Miao[1,#], T. T. Zhang[2], H. X. Li[1,3], G. Fabbris[4], A. H. Said[4], R. Tartaglia[4,5], T. Yilmaz[6], E. Vescovo[6], J.-X. Yin[7], S. Murakami[2], L. X. Feng[8], K. Jiang[8], X. L. Wu[9], A. F. Wang[9,#], S. Okamoto[1,#], Y. L. Wang[10,#], H. N. Lee[1]

[1]*Materials Science and Technology Division, Oak Ridge National Laboratory, Oak Ridge, Tennessee 37831, USA*

[2]*Department of Physics, Tokyo Institute of Technology, Okayama, Meguro-ku, Tokyo 152-8551, Japan*

[3]*Advanced Materials Thrust, The Hong Kong University of Science and Technology (Guangzhou), Guangzhou, Guangdong 511453, China.*

[4]*Advanced Photon Source, Argonne National Laboratory, Argonne, Illinois 60439, USA*

[5]*"Gleb Wataghin" Institute of Physics, University of Campinas, Campinas, São Paulo 13083-859, Brazil*

[6]*National Synchrotron Light Source II, Brookhaven National Laboratory, Upton, New York 11973, USA*

[7]*Laboratory for Quantum Emergence, Department of Physics, Southern University of Science and Technology, Shenzhen, Guangdong 518055, China.*

[8]*Beijing National Laboratory for Condensed Matter Physics, and Institute of Physics, Chinese Academy of Sciences, Beijing 100190, China*

[9]*Low Temperature Physics Laboratory, College of Physics and Center of Quantum Materials and Devices, Chongqing University, Chongqing 401331, China*

[10]*School of Emerging Technology, University of Science and Technology of China, Hefei, Anhui 230026, China*

Correspondence should be addressed to:

miaoh@ornl.gov, afwang@cqu.edu.cn, okapon@ornl.gov, and yilinwang@ustc.edu.cn.

Supplementary Figure 1: Transport and susceptibility of FeGe.
Supplementary Figure 2: Fermi surface topology and ARPES intensity plots along high-symmetry directions.
Supplementary Figure 3: HKL-scans at CDW wavevectors.
Supplementary Figure 4: Phonon dynamical structure factor along high-symmetry directions and the absence of Kohn-anomaly at $Q_M^{//}$ and Q_L
Supplementary Figure 5: Atoms vibrations of the B_{1u} phonon modes at the three equivalent L-points.
Supplementary Figure 6: Calculated electronic band structures of FeGe in AFM phase.
Supplementary Figure 7: DFT+U calculated 2×2×2 superstructure in FeSn.
Supplementary Figure 8: The DFT-calculated charge density map of the CDW ground state.
Supplementary Figure 9: Schematic view of the theoretical model of phonon-magnon coupling.
Supplementary Figure 10. Second-order Feynman diagram for the phonon self-energy $\Sigma(q, i\omega_n)$.
Supplementary Figure 11: Numerical results of the phonon self-energy $\Sigma(q, \omega)$and Green's function $D(q, \omega)$
Supplementary Discussion: Phonon lifetime by two magnon excitations.

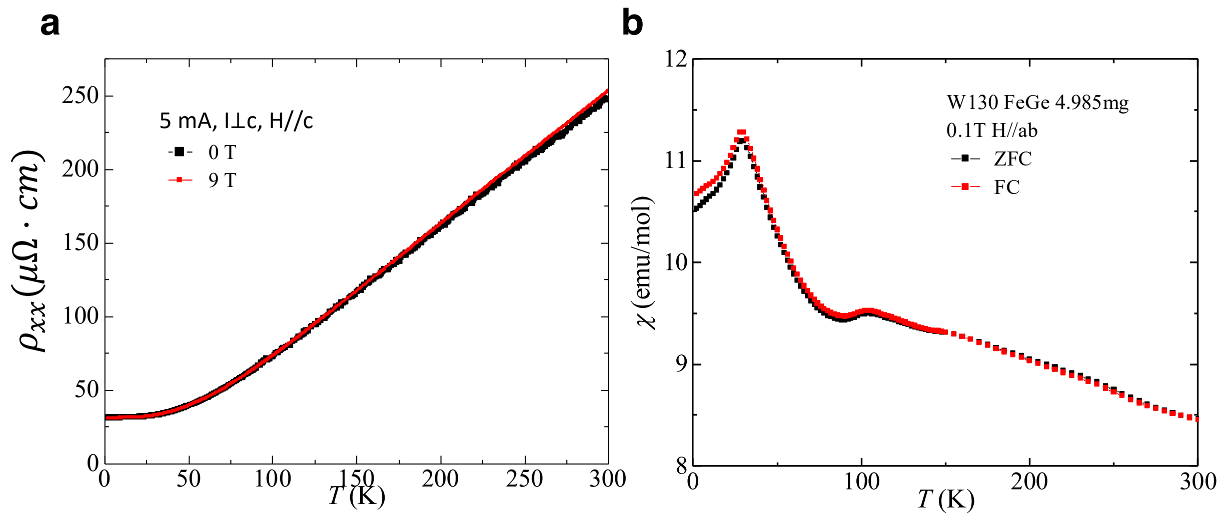

Supplementary Figure 1: Transport and susceptibility of FeGe. a, temperature dependent in-plane resistivity at H=0 T (black) and H=9 T (red). **b,** temperature dependent magnetic susceptibility. Black and red curves represent zero-field cooling (ZFC) and field-cooling (FC), respectively. The hump slightly above 100 K is corresponding to the onset of charge-dimerization and charge density wave.

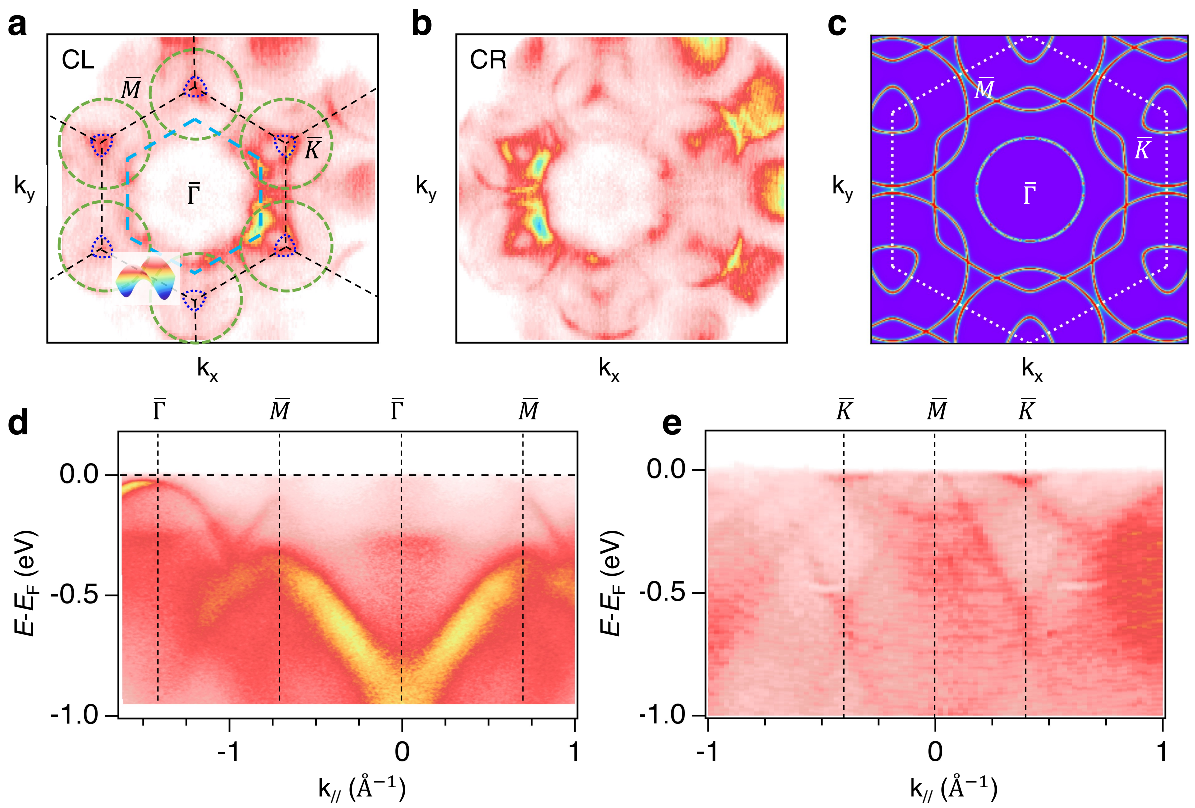

Supplementary Figure 2: Fermi surface topology and ARPES intensity plots along high-symmetry directions. a and **b** are Fermi surface mappings of FeGe measured by circular left (CL) and circular right (CR) incident photon polarization, respectively. The data were taken at photon energy $h\nu$=100 eV. The surface high symmetry points are defined in Fig.1 of the main text. **c,** DFT+DMFT calculated Fermi surface at k_z=0 in the A-type AFM phase at U=4.2 eV and J_H=0.88 eV shows good consistence with experimental data. **d** and **e**, ARPES intensity plots along the high symmetry $\bar{\Gamma}$- $\bar{M}$ and $\bar{K}$- $\bar{M}$ direction, respectively. In agreement with previous study, the van Hove singularity at the $\bar{M}$ point is slightly above the Fermi level[24]. The overall Fermi surface topology and band structure are very similar to FeSn[3], challenging a weak-coupling picture based on van Hove singularity for CDW in FeGe.

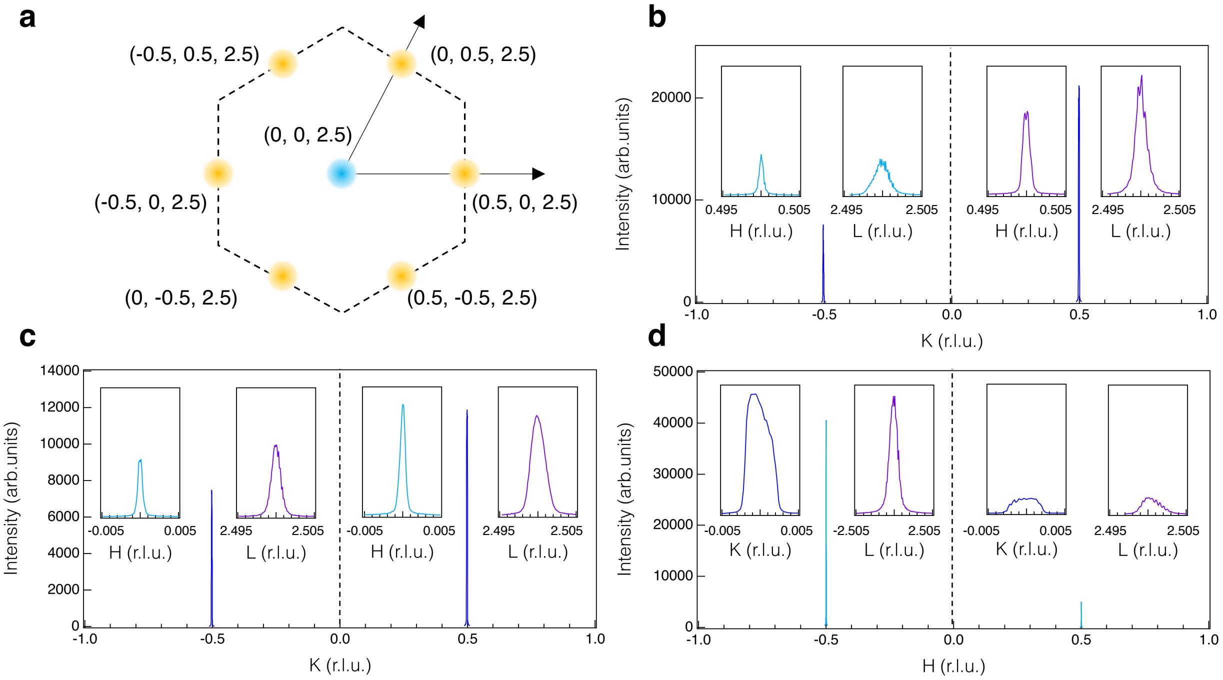

Supplementary Figure 3: HKL-scans at CDW wavevectors. Cyan, Blue and purple curves are corresponding to *H*, *K* and *L* scans respectively. **a**, CDW wavevectors (yellow) and Kagome dimer vector (Blue) at the *L*=2.5 plane in the momentum space. **b**, shows *HKL*-scans at **Q**=(0.5, -0.5, 2.5) and (-0.5, 0.5, 2.5). **c**, shows *HKL*-scans at **Q**=(0, -0.5, 2.5) and (0, 0.5, 2.5). **d**, shows *HKL*-scans at **Q**=(0.5, 0, 2.5) and (-0.5, 0, 2.5). Note *K*- and *L*-scans are broader than H-scans. This is due to the slightly worse momentum resolution for the *K*- and *L*-scans in our experimental geometry.

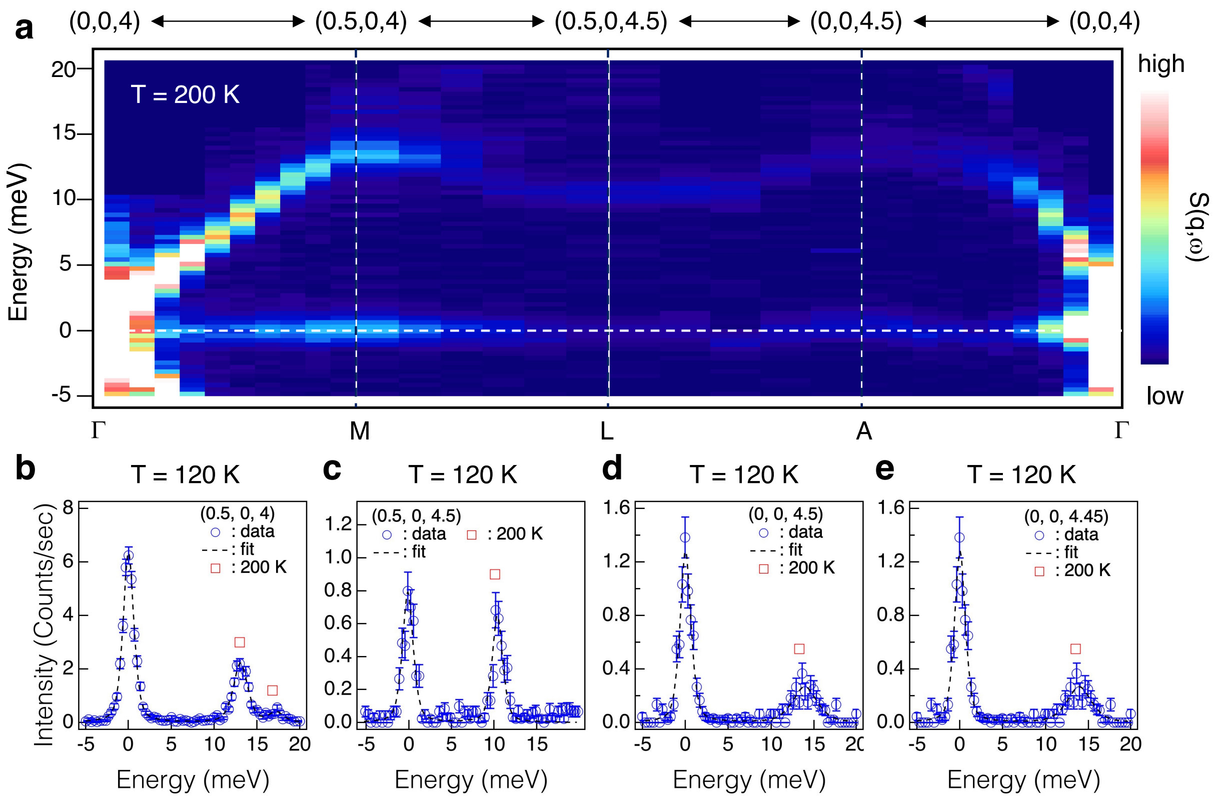

Supplementary Figure 4: Phonon dynamical structure factor along high-symmetry directions and the absence of Kohn-anomaly at $Q_M^{//}$ and Q_{L}**. a,** experimental S(Q, ω) along the **Γ**(0, 0, 4)-**M**(0.5 ,0, 4)-**L**(0.5, 0, 4.5)-**A**(0, 0, 4.5)-**Γ**(0, 0, 4) direction determined by inelastic x-ray scattering at 200 K. **b-e**, IXS spectra at ***Q***=(0.5, 0, 4), (0.5, 0, 4.5), (0, 0, 4.5) and (0, 0, 4.45). These curves were taken at 120 K, slightly above T_{CDW} to avoid strong elastic intensity from CDW and charge dimer. Dashed black curves are fittings of the experimental data (see Methods). The red squares shown in **b-e** are the extracted phonon peak positions at 200 K from the same Qs and the same phonon modes. The lateral size of the red squares represents the instrumental energy resolution. The vertical error bars in panel **b-e** represent 1-standard deviation assuming Poisson counting statistics.

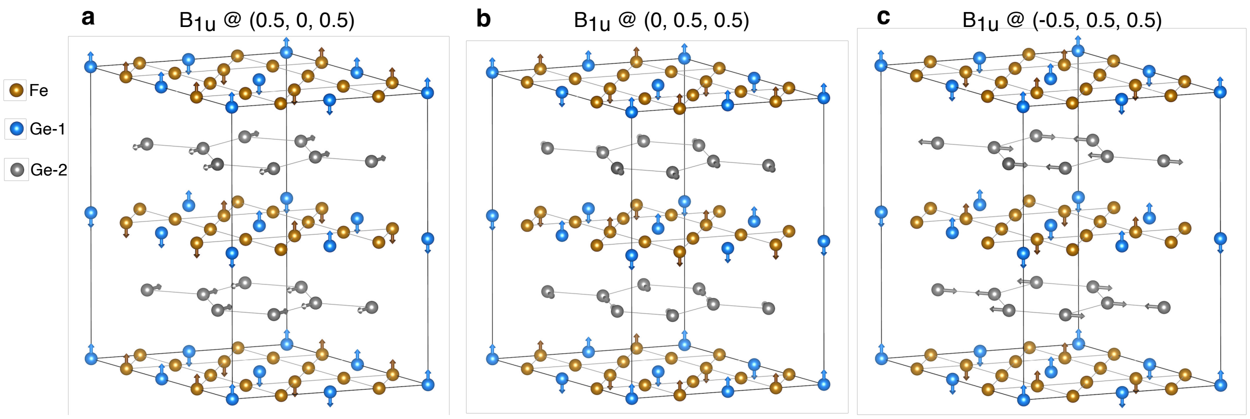

Supplementary Figure 5: Atoms vibrations of the B_{1u} phonon modes at the three equivalent L-points. a,**b**,**c,** for L points (0.5, 0, 0.5), (0, 0.5, 0.5), (-0.5, 0.5, 0.5), respectively. The arrows indicate movements of Fe and Ge atoms. In addition to the $2 \times 2 \times 2$ superstructure, we have also employed other lattice distortion ansätze, including (i)$1 \times 2 \times 2$ superstructure, corresponding to any one of the three B1u mode at L-point; (ii) $\sqrt{3} \times \sqrt{3} \times 2$ superstructure corresponding to the B1u phonon mode at H-point; and (iii) a $\sqrt{5} \times \sqrt{5} \times 2$ superstructure. All these ansätze yield ground state energies higher than the $2 \times 2 \times 2$ superstructure and the original ideal Kagome structure. This indicates that the $2 \times 2 \times 2$ superstructure optimized by DFT is indeed the true ground state even though ΔE for small U is close to zero. This small energy difference at small U may be consistent with experimental observation where both the CDW transition temperature and correlation length can be reduced significantly by post annealing process[51].

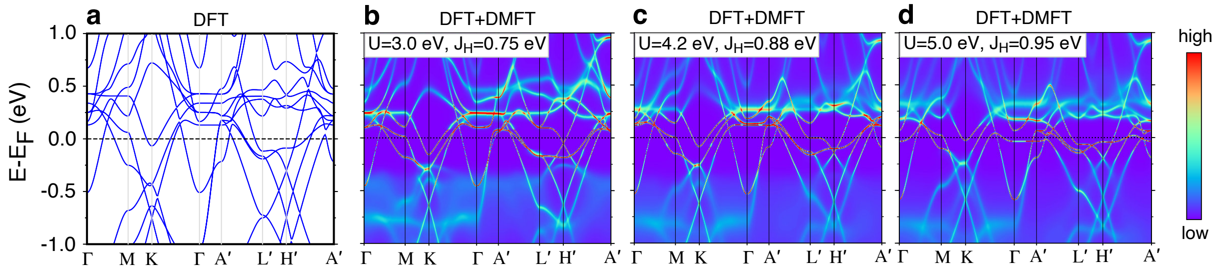

Supplementary Figure 6: Calculated electronic band structures of FeGe in AFM phase. a, Electronic band structures calculated by DFT. **b, c**, **d**, Electronic spectra functions calculated by DFT+DMFT at $U = 3\ eV, J_H = 0.75\ eV$; $U = 4.2\ eV, J_H = 0.88\ eV$ and $U = 5\ eV, J_H = 0.95\ eV$, respectively. The van-Hove singularities are located at M point near the Fermi level. Here, the band structures are plotted with respect to the AFM BZ.

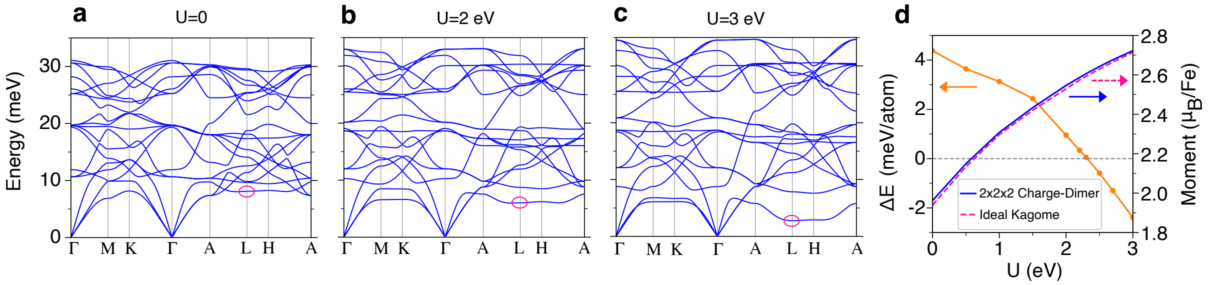

Supplementary Figure 7: Spin-polarization-induced 2×2×2 superstructure in FeSn. As we show in Fig. S2, the Fermi surface topology is very similar between the isostructure FeSn and FeGe. Indeed, FeSn also features A-type AFM with a T_N~365 K[36]. Therefore, it is important to understand why FeSn fails to stabilize the 2×2×2 superstructure. **a,b,c**, Similar to FeGe, the calculated phonon spectra of FeSn also show dramatic change of the B_{1u} phonon at L-point as increasing U, as marked by red circles. **d**, Left y-axis shows the energy difference between the locally "charge-dimerized" $2 \times 2 \times 2$ superstructure and the ideal Kagome phase, $\Delta E = E_{Charge-Dimer} - E_{Kagome}$. In contrast to FeGe, the charge dimerized superstructure has lower energy only at very large U (>2.4 eV), suggesting that FeSn favors a ground state with ideal Kagome structure. Possible reason for the difference between these compounds is that the c-axis lattice parameter of FeSn is about 10% larger than that of FeGe, which makes the dimerization along c-axis unfavorable in FeSn. Indeed, the spin polarization enhancement in FeGe is about twice larger than FeSn for U<1 eV.

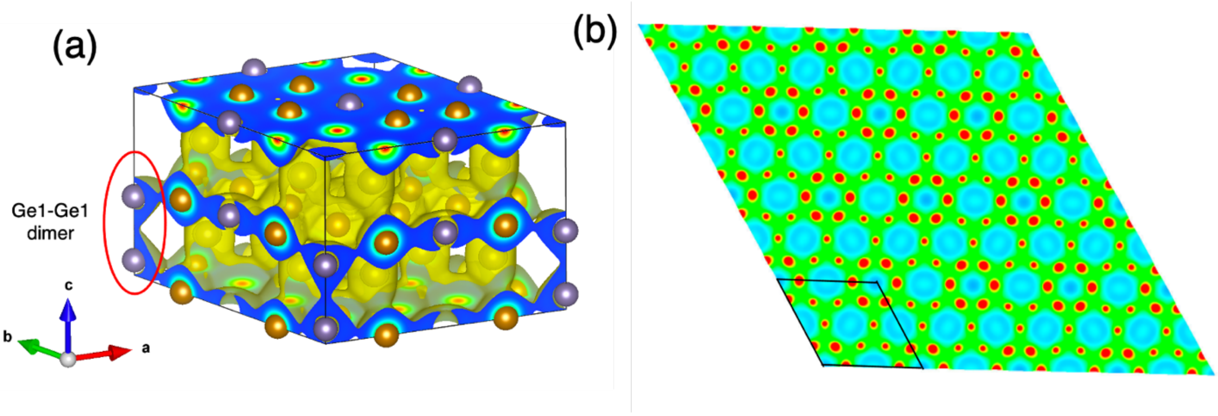

Supplementary Figure 8: The DFT-calculated charge density map of the CDW ground state. **a,** 3D visualization of charge distribution in the CDW phase. Red ellipse highlights the Ge1-Ge1 dimer. **b**, top view of the charge distribution in the kagome plane.

Supplementary Discussion: Phonon lifetime by two magnon excitations

We now discuss how spin-phonon coupling induces phonons lifetime effect. Longitudinal lattice displacements modify exchange coupling between neighboring spins. In an antiferromagnet, such ionic displacements could excite two magnons, one has, say, right helicity and another has left helicity and therefore the total spin remains unchanged. Suppose a phonon has momentum q and energy ω_q decay into two magnons, one with momentum k and energy ε_k and another with momentum k' and energy $\varepsilon_{k'}$. Because of the energy and momentum conservation, $\varepsilon_k + \varepsilon_{k'} = \omega_q$ and $k + k' = q$ have to be satisfied. This leads to the condition $\varepsilon_k + \varepsilon_{q-k} = \omega_q$, which means there is minimal energy ω_q of phonons to decay into two magnons. Furthermore, because longitudinal phonon modes at small momentum q consist of nearly uniform displacement of lattice sites and, therefore, do not modify exchange coupling. To couple with magnons, longitudinal phonons need to have large momentum.

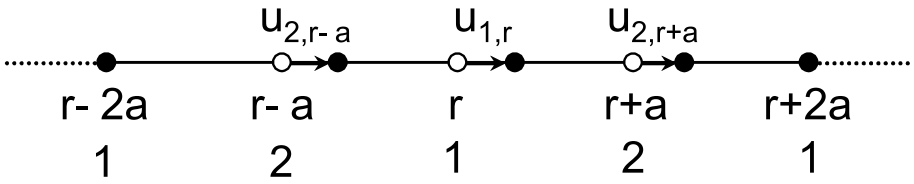

Figure 9. Schematic view of the theoretical model of phonon-magnon coupling. Displacement of magnetic ions is given by $u_{l,r}$. Such displacements modify exchange coupling between neighboring spins and excite magnons. The lattice constant a is be taken to be unity.

To demonstrate the coupling between longitudinal phonons and magnons, here we consider a one-dimensional antiferromagnetic Heisenberg chain, which captures the essential physics of magnetism and phonons in FeGe along the z direction. Assuming a Néel ordering with two magnetic sublattices, 1 and 2, such coupling could be modeled by:

$$H_{pm} = \frac{-1}{2} g \sum_{r \in sublattice1} \left[\left(u_{1,r} - u_{2,r+a}\right)\left(\alpha^{\dagger}_{1,r}\alpha^{\dagger}_{2,r+a} + \alpha_{1,r}\alpha_{2,r+a}\right) + \left(u_{2,r-a} - u_{1,r}\right)\left(\alpha^{\dagger}_{2,r-a}\alpha^{\dagger}_{1,r} + \alpha_{2,r-a}\alpha_{1,r}\right)\right]. \quad (1)$$

Here, g is the phonon-magnon coupling constant, representing the modulation of exchange by atomic displacements, $u_{l,r}$is a longitudinal displacement of the lattice site at sublattice l at position r. a is the lattice constant, which will be taken to be unity. $\alpha^{(\dagger)}_{l,r}$ is a magnon annihilation (creation) operator at sublattice l at position r. For simplicity, we only consider the Fourier transform of magnon annihilation and creation operators as $\alpha_{l,r} = \sqrt{\frac{2}{N}} \sum_k e^{ikr}\, \alpha_{l,k}$ and $\alpha^{\dagger}_{l,r} = \sqrt{\frac{2}{N}} \sum_k e^{-ikr}\, \alpha^{\dagger}_{l,k}$, respectively, where, N is the number of lattice sites. $\alpha_{l,k}$ and $\alpha^{\dagger}_{l,k}$ dagonalize the magnon Hamiltonian as:

$$H_m = \sum_k \varepsilon_k \left(\alpha^{\dagger}_{1,k}\alpha_{1,k} + \alpha^{\dagger}_{2,k}\alpha_{2,k}\right) \qquad (2)$$

Here, $\varepsilon_k = 2J\sqrt{(1+d)^2 - \cos^2 k}$ is the magnon dispersion, with J the exchange constant, which will be taken as the unit of energy below, and d represents the single-ion anisotropy along the z axis. The two magnon modes, $l = 1$ and $l = 2$, have opposite helicities.

Introducing the Fourier transform of the lattice displacement fields as $u_{l,r} = \sqrt{\frac{2}{N}} \sum_q e^{-iqr}\, u_{l,q}$,we arrive at

$$H_{pm} = -gi\sqrt{\frac{2}{N}} \sum_{q,k} \left\{ \sin(q+k) u_{1,q} - \sin k u_{2,q} \right\} \alpha^{\dagger}_{1,k}\alpha^{\dagger}_{2,-k-q} + H.c. \quad (3)$$

Note that the sum of momenta, q and k, is limited in the magnetic Brillouin zone $[-\pi/2, \pi/2]$. Now, we separate the displacement fields between acoustic and optical modes, $u_{a,q}$ and $u_{o,q}$, respectively as $u_{a,q} = \frac{1}{\sqrt{2}}\left(u_{1,q} + u_{2,q}\right)$ and $u_{o,q} = \frac{1}{\sqrt{2}}\left(u_{1,q} - u_{2,q}\right)$. This leads to:

$$H_{pm} = -gi\sqrt{\frac{1}{N}}\sum_{q,k}\left[\{\sin(q+k) - \sin k\}u_{a,q} + \{\sin(q+k) + \sin k\}u_{o,q}\right]\alpha_{1,k}^{\dagger}\alpha_{2,-k-q}^{\dagger} + H.c. \quad (4)$$

Finally, by second quantizing the displacement fields as $u_{a,q} = \sqrt{\frac{1}{2M\omega_{a,q}}}\left(a_q^{\dagger} + a_{-q}\right)$ and $u_{o,q} = \sqrt{\frac{1}{2M\omega_{o,q}}}\left(o_q^{\dagger} + o_{-q}\right)$, where $a_q^{(\dagger)}$ and $o_q^{(\dagger)}$ are corresponding annihilation (creation) operators of longitudinal phonons, the phonon-magnon coupling is written as:

$$H_{pm} = -g\sqrt{\frac{1}{N}}\sum_{q,k} i\left[\sqrt{\frac{1}{2M\omega_{a,q}}}\{\sin(q+k) - \sin k\}\left(a_q^{\dagger} + a_{-q}\right) + \sqrt{\frac{1}{2M\omega_{o,q}}}\{\sin(q+k) + \sin k\}\left(o_q^{\dagger} + o_{-q}\right)\right]\alpha_{1,k}^{\dagger}\alpha_{2,-k-q}^{\dagger} + H.c. \quad (5)$$

Here, M is the mass of a magnetic ion, $\omega_{a,q}$ and $\omega_{o,q}$ are the dispersion of acoustic and optical phonons given by $\omega_{a,q} = \sqrt{\frac{2K}{M}(1-\cos q)} = \sqrt{\frac{4K}{M}}\left|\sin\left(\frac{q}{2}\right)\right|$ and $\omega_{o,q} = \sqrt{\frac{2K}{M}(1+\cos q)} = \sqrt{\frac{4K}{M}}\left|\cos\left(\frac{q}{2}\right)\right|$, respectively, where K is the spring constant. We then focus on the acoustic mode and extend the momentum q space to $[-\pi, \pi]$. This leads to:

$$H_{pm} = -g\sqrt{\frac{1}{N}}\sum_{q,k} i\sqrt{\frac{1}{2M\omega_q}}\{\sin(k-q) - \sin k\}\left(a_q^{\dagger} + a_{-q}\right)\alpha_{1,k}^{\dagger}\alpha_{2,q-k}^{\dagger} + H.c. \quad (6)$$

where the subscript of the phonon dispersion is removed for simplicity as $\omega_q = \omega_{a,q,}$.

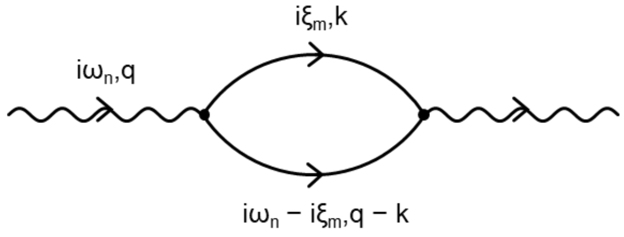

Figure 10. Second-order Feynman diagram for the phonon self-energy $\Sigma(q, i\omega_n)$. Solid lines are the magnon Green's function. $i\omega_n$ and $i\xi_m$ are bosonic Matsubara frequencies.

The phonon self-energy is computed by the second-order perturbation using the Matsubara formalism. The self-energy is schematically shown in Fig. S6, and its analytic form is given by:

$$\Sigma(q, i\omega_n) = \frac{g^2}{8\sqrt{KM}}\frac{2T}{N}\sum_{i\xi_m,k}\frac{[\sin(k-q)-\sin k]^2}{|\sin(q/2)|}\frac{1}{i\omega_n - i\xi_m - \varepsilon_{q-k}}\frac{1}{i\xi_m - \varepsilon_k} \quad (7)$$

where $i\omega_n$ and $i\xi_m$ are bosonic Matsubara frequencies given by $i\omega_n = 2n\pi Ti$ and $i\xi_m = 2m\pi Ti$, respectively, with n and m begin integers. After the summation over the Matsubara frequency $i\xi_m$ and the analytic continuation to real frequency as $i\omega_n \to \omega + i\eta$, where $i\eta$ is a small imaginary number, one obtains the phonon retarded self-energy at zero temperature as:

$$\Sigma(q, \omega) = \frac{g^2}{8\sqrt{KM}}\frac{2}{N}\sum_{k}\frac{[\sin(k-q)-\sin k]^2}{|\sin(q/2)|}\frac{1}{\omega - \varepsilon_k - \varepsilon_{q-k} + i\eta} \quad (8)$$

As mentioned previously, phonon-magnon coupling should disappear at $q \to 0$ for longitudinal phonons. One can confirm this behavior by noticing $\frac{[sin(k-q)-sink]^2}{|sin(q/2)|} 2|q|cos^2 k$ at small q. Thus, $\Sigma(q,\omega)$ indeed becomes zero at $q = 0$. With this self-energy, the phonon retarded Green's function is given by:

$$D(q, \omega) = \frac{1}{\omega - \omega_q - \Sigma(q,\omega) + i\eta} \quad (9)$$

Supplementary Figure 11 summarizes the results of the present analysis using two sets of magnon dispersions, one without single-site anisotropy $d = 0$ (a-c) and one with anisotropy $d = 0.05$ (d-f). Here, the intensity of the phonon self-energy is scaled by $g^2/8\sqrt{KM}$. To compute the Green's function $D(q,\omega)$, phonon-magnon coupling $g^2/8\sqrt{KM} = 0.2$ and the bare band width of phonon dispersions $2\sqrt{K/M} = 3$ are used. When $d = 0$, magnons have gapless excitations at $k = 0$, i.e., $\varepsilon_0 = 0$. Therefore, the continuum of the imaginary part of the phonon self-energy has a lower bound set by magnon excitation energy ε_k because the energy and momentum conservation $\omega_q = \varepsilon_q$ is always satisfied [see Fig. 11 (a)]. With nonzero d, $\varepsilon_0 = 2J\sqrt{(2+d)d}$, therefore $\omega_q > \varepsilon_q$ and there is a gap ε_0 between the continuum and magnon dispersion ε_q [Fig. 11 (d)]. The real part of

the self-energy is related to the imaginary part by the Kramers–Kronig relation. Therefore, it has a negative peak near the lower bound of the continuum of the imaginary part and a positive peak slightly above the lower bound [Figs. 11 (b,e)]. Obviously, the continuum of the imaginary part of the self-energy has upper bound $2\varepsilon_{q=\pi/2}$ at $q=\pi$. The phonon Green's function reflects these behaviors of the self-energy. When phonon dispersion ω_q is below the continuum, the phonon spectral function has a well-defined peak at $\omega=\omega_q+\Re\Sigma(q,\omega_q)$, and when ω_q hits the continuum, the phonon spectral function rapidly loses its intensity [Figs. 11 (c,f)].

While magnon dispersions in FeGe have not been reported yet, based on the current theoretical analysis, we speculate that longitudinal phonons decay into two magnons at near the A point, experiencing the sudden broadening of the spectral function. We shall note that our analysis is based on a simplified 1D spin-chain and hence serves as a proof-of-principle analysis. In FeGe, as we have shown in the main text, the complicated lattice distortion is expected to give rise more complicated spin-phonon coupling and phonon self-energy effects.

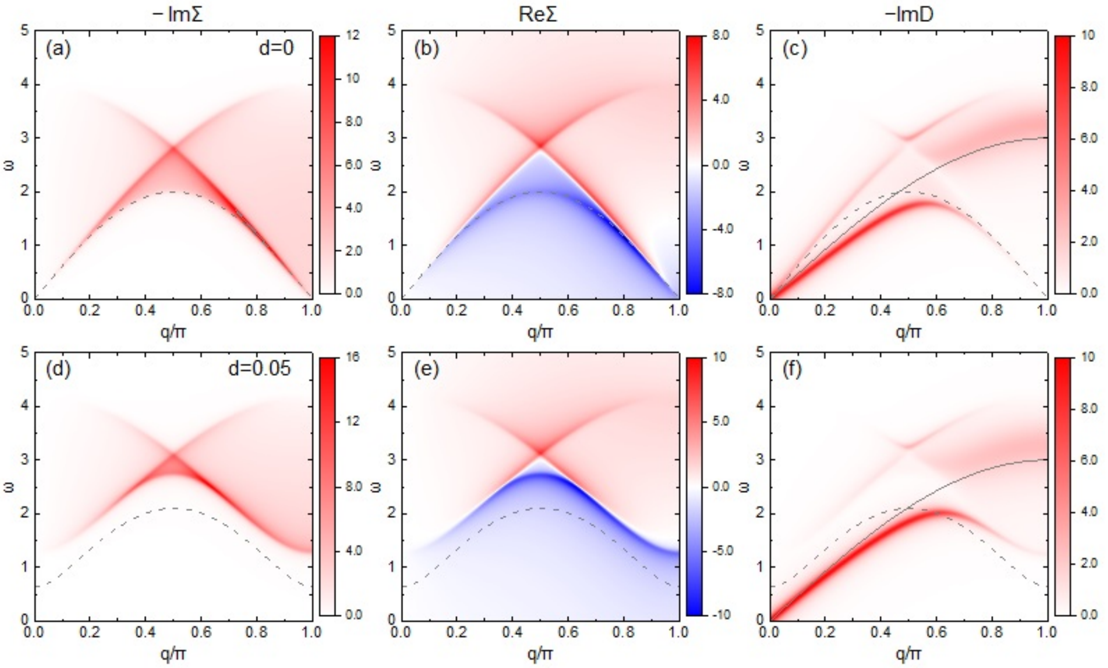

Figure 11: Numerical results of the phonon self-energy $\Sigma(q,\omega)$and Green's function $D(q,\omega)$. a-c, $d=0$ and d-f, $d=0.05$. (a,d) The Imaginary part of $\Sigma(q,\omega)$, (b,e) the real part of $\Sigma(q,\omega)$, and (c,f) the imaginary part of $D(q,\omega)$. The intensity of the phonon self-energy is scaled by

$g^2/8\sqrt{KM}$. For $D(q,\omega)$, phonon-magnon coupling $g^2/8\sqrt{KM} = 0.2$ and the bare band width of phonon dispersions $2\sqrt{K/M} = 3$ are used. For numerical calculations, a small imaginary number $i\eta$ is introduced with $\eta = 0.05$. Dashed curves are magnon dispersions, and solid lines are bare phonon dispersions.
